# Azacitidine Is Synergistically Lethal with XPO1 Inhibitor Selinexor in Acute Myeloid Leukemia by Targeting XPO1/eIF4E/c-MYC Signaling

**DOI:** 10.3390/ijms24076816

**Published:** 2023-04-06

**Authors:** Huideng Long, Yue Hou, Jun Li, Chunhua Song, Zheng Ge

**Affiliations:** 1 Department of Hematology, Zhongda Hospital, School of Medicine, Southeast University, Institute of Hematology Southeast University, Nanjing 210009, China; 2 Hershey Medical Center, Pennsylvania State University Medical College, Hershey, PA 17033, USA; 3 Division of Hematology, The Ohio State University Wexner Medical Center, The James Cancer Hospital, Columbus, OH 43210, USA

**Keywords:** XPO-1 inhibitor, azacitidine, acute myeloid leukemia, apoptosis, eIF4E, c-MYC

## Abstract

Acute myeloid leukemia (AML) is a high-mortality malignancy with poor outcomes. Azacitidine induces cell death and demonstrates treatment effectiveness against AML. Selinexor (KPT-330) exhibited significant benefits in combination with typical induction treatment for AML patients. Here, we explore the antitumor effect of KPT-330 combined with AZA in AML through CCK-8, flow cytometry, RT-qPCR, western blot, and RNA-seq. Our results showed that KPT-330 combined with AZA synergistically reduced cell proliferation and induced apoptosis in AML primary cells and cell lines. Compared to the control, the KPT-330 plus AZA down-regulates the expression of XPO1, eIF4E, and c-MYC in AML. Moreover, the knockdown of c-MYC could sensitize the synergy of the combination on suppression of cell proliferation and promotion of apoptosis in AML. Moreover, the expression of *XPO1* and *eIF4E* was elevated in AML patient cohorts, respectively. *XPO1* and *elF4E* overexpression was associated with poor prognosis. In summary, KPT-330 with AZA exerted synergistic effects by suppressing XPO1/eIF4E/c-MYC signaling, which provided preclinical evidence for further clinical application of the novel combination in AML.

## 1. Introduction

Acute myeloid leukemia (AML) is a devastating hematologic malignancy characterized by a group of clonal diseases with high heterogeneity, and its incidence has increased with age in recent years [1,2,3,4]. Only 35% to 40% of adult AML patients younger than 60, while only 5% to 15% of AML patients over 60 experienced clinical remissions [5,6,7,8]. The efficacy of current clinical therapies is still far satisfactory for AML patients, and new therapeutic strategies are always urgently needed.

Epigenetic abnormalities and gene silencing caused by hypermethylation of CpG islands, the promoter region of tumor-associated genes, could exert crucial effects in the pathogenesis of AML [9,10]. The reversal of abnormal gene methylation and the promotion of tumor suppressor gene re-expression is the primary evidence for treating AML with demethylating drugs, including azacitidine (AZA) [11,12]. AZA can delay the transformation of myelodysplastic syndromes (MDS) patients to AML and improve patients’ blood transfusion dependence and quality of life, thus, becoming one of the main treatment options for MDS and AML [13,14]. However, clinical observations showed that not all patients benefited from AZA therapy, and some patients may become ineffective or resistant to AZA monotherapy [15,16,17,18]. Therefore, it is significant to explore the combination of AZA in treating AML.

Exportin 1 (XPO1) is one of the major players in protein transport in the nucleus, and recently it is also observed that XPO1 alteration plays a vital role in tumor pathogenesis [19]. XPO1 is the only exporter of several tumor suppressor and cell cycle regulator proteins, such as p53, FOXO, PTEN, and NF-kB [20]. XPO1 is highly expressed and associated with poor prognosis in hematological malignancies [21,22,23,24]. XPO1 inhibitor has emerged as an approach for treating cancer. Selinexor (KPT-330) is a new XPO1 inhibitor and is an effective small-molecule drug with promising antitumor effects in various human tumors, including AML in vitro and in vivo [23,25,26,27]. Several studies indicated that selinexor monotherapy [28,29] or combination therapy shows promising clinical activity and safety for the elderly and relapsed or refractory (R/R) AML patients [30,31,32]. Due to its effectiveness, novel combinations of KPT-330 with AZA (NCT05736965, NCT05736978) were under clinical investigation. However, it is undetermined about the effect and the underlying mechanism of KPT-330 combined with AZA in AML.

In this study, the synergistic effect of KPT-330 with AZA on the reduction of cell proliferation and promotion of apoptosis in AML cells was observed, and the molecular mechanism of the XPO1/eIF4E/c-MYC axis was determined through global transcriptome analysis.

Our results indicate that combining KPT-330 with AZA is a new potential therapeutic strategy in AML and provides preclinical evidence for further clinical trials. Based on the present data, a clinical trial of KPT-330 plus AZA in AML is under registration and will be performed.

## 2. Results

### 2.1. Synergistic Effect of KPT-330 Combined with AZA on Cell Proliferation of AML Cells

**Figure 1:**
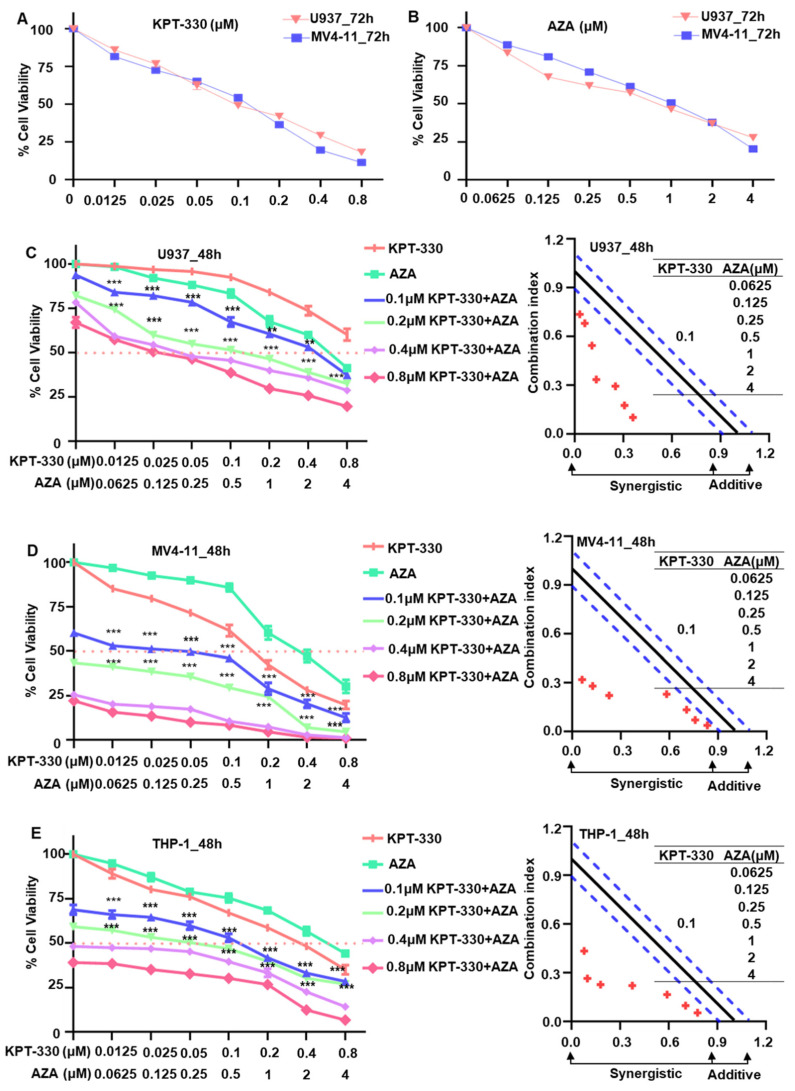
KPT-330 combined with AZA synergistically inhibited AML cell growth. (**A**) Cell proliferation assay result (dose-dependent curve) for KPT-330 in U937 and MV4-11 for 72 h. (**B**) Cell proliferation assay result (dose-dependent curve) for AZA in U937 and MV4-11 for 72 h. (**C**) Synergistic effect of KPT-330 with various doses of AZA on cell proliferation arrest and CalcuSyn analysis in U937 cells. (**D**) Synergistic effect of KPT-330 with various doses of AZA on cell proliferation arrest and CalcuSyn analysis in MV4-11 cells. (**E**) Synergistic effect of KPT-330 with various doses of AZA on cell proliferation arrest and CalcuSyn analysis in THP-1 cells. “……” indicates IC50, “----” indicates the range of synergies. ** *p* < 0.01, *** *p* < 0.001.

To observe the effect of KPT-330 or AZA on cell proliferation of AML cells, U937, MV4-11, and THP-1 cells were administered with varying doses of KPT-330 (0–0.8 μM) and AZA (0–4 μM) for 24, 48, and 72 h (Figure S1A–D and Figure 1A,B). Results showed that treatment of KPT-330 or AZA significantly induced the cell proliferation arrest in U937 and MV4-11 cells in a time- and dose-dependent manner (Figure S1A–D and Figure 1A,B), and similar results were acquired in THP-1 cell lines (Figure S1A–D). The IC50 was evaluated in AML cell lines treated for 24–72 h with KPT-330 or AZA (Figure S1E,F).

To evaluate the synergistic impact of KPT-330 in combination with AZA on AML cells, we processed cells with varying concentrations of AZA in conjunction with a constant concentration of KPT-330. All fixed dosages of KPT-330 (0.1 μM, 0.2 μM, 0.4 μM, 0.8 μM) significantly enhanced the impact of AZA on the restriction of cell growth in U937, MV4-11, and THP-1 cells (Figure 1C–E). CalcuSyn analysis showed the synergism of KPT-330 combined with AZA on proliferation inhibition of AML cells (Figure 1C–E). ZIP analysis with Loewe and Bliss modeling also showed significant synergistic effects and synergistic doses of KPT-330 and AZA in AML cells (Figure S2). Overall, the above data demonstrated that the combination of KPT-330 and AZA synergistically inhibits AML cell growth.

### 2.2. Synergistic Effect of KPT-330 with AZA on Apoptosis of AML Cells

**Figure 2:**
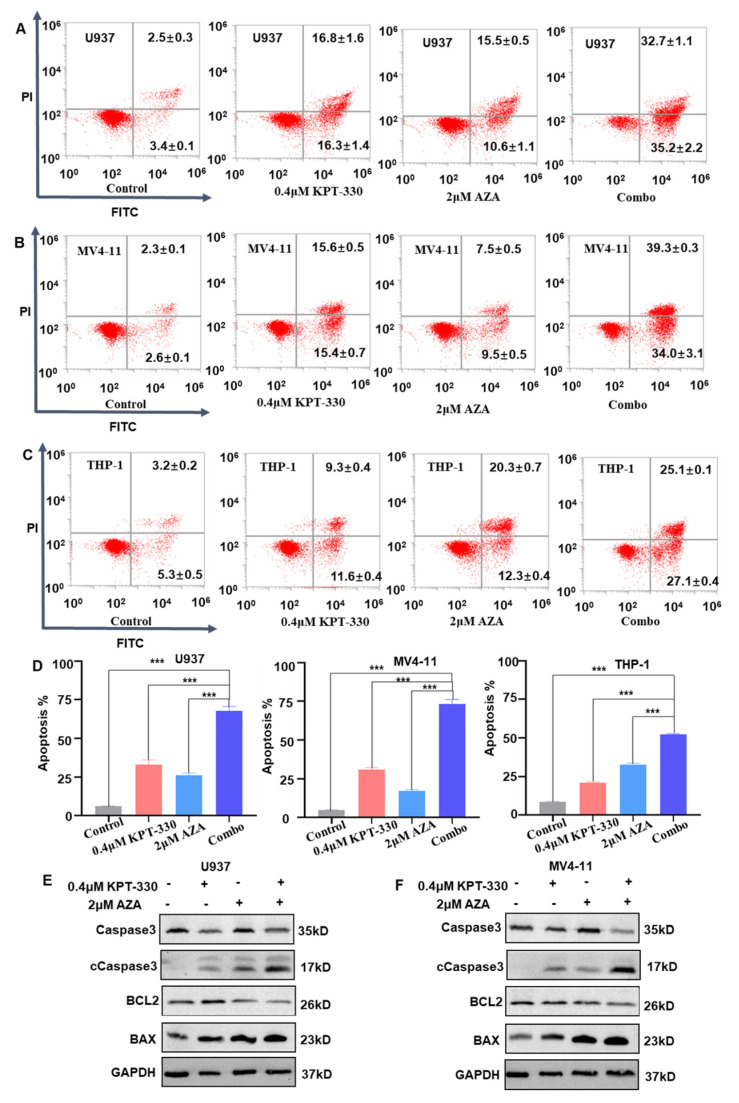
Synergistic effect of KPT-330 with AZA on apoptosis of AML cells. (**A**) Effect of KPT-330 with AZA on apoptosis of U937 cells. (**B**) Effect of KPT-330 with AZA on apoptosis of MV4-11 cells. (**C**) Effect of KPT-330 on apoptosis of THP-1 cells. (**D**) Quantitative bar charts for the three cell lines. (**E**,**F**) Western blot of the apoptotic proteins (Caspase3, cleaved Caspase3 (cCaspase3), BCL2, and BAX) in the U937 and MV4-11 cells upon the drug treatment (DMSO, KPT-330, AZA, and combo). *** *p* < 0.001.

To examine the effect of KPT-330 with AZA on apoptosis of AML cells, we treated cells with DMSO vehicle control, 0.4 μM KPT-330, 2 μM AZA, and combo, respectively. We also treated cells with DMSO vehicle control, 0.2 μM KPT-330, 1 μM AZA, and combo in the MV4-11 cell line (Figure S3A). Results suggested that the combination of KPT-330 with AZA showed a significantly higher apoptotic rate in U937, MV4-11, and THP-1 cells compared to either each single drug control (Figure 2A–D). The pro-apoptotic BAX protein and cleaved Caspase3 were consistently enhanced, while the anti-apoptotic BCL-2 protein and Caspase3 were reduced upon the combination treatment compared to single drug control (Figure 2E,F). The mRNA level of the apoptosis-related genes (*BCL2*, *BAX*, and *DDIT3*) showed a similar change upon the drug treatment in U937, MV4-11, and THP-1 cells (Figure S4).

In order to explore the synergistic effects of KPT-330 with AZA, we performed a cell cycle analysis using a DMSO vehicle control, 0.4 μM KPT-330, 2 μM AZA, and a combination of the two in U937 and MV4-11 cells. As shown in Figure S3B–D, combination treatment induced a significant cell cycle arrest in the G1 phase in U937 compared to KPT-330 or AZA monotherapy, but we observed different patterns of cell cycle arrest among the various AML cell lines. In particular, we did not find a significant upregulation in G1 phase arrest in the combo group in MV4-11 cells when compared to mono-treatment. These findings suggest that cell cycle arrest may not be the primary mechanism underlying the synergistic effect of the combination KPT-330 and AZA (Figure S3B–D).

### 2.3. Transcriptome Analysis to Identify the Key Genes and Pathway Responsible for the Synergistic Effect

**Figure 3:**
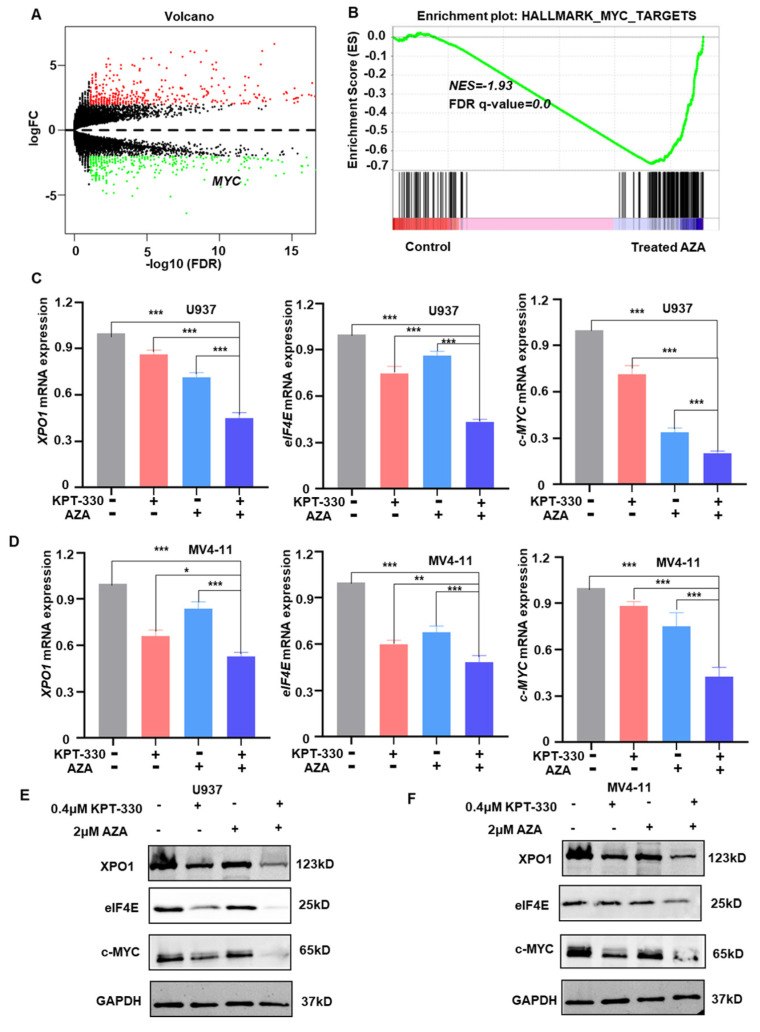
Transcriptome analysis to identify the key genes and pathways responsible for the synergistic effect. (**A**) RNA-seq data volcano graph; Red represents up-regulated genes and green represents down-regulated genes. (**B**) GSEA analysis graph. (**C**,**D**) qPCR data to show the changes of the key genes (*XPO1*, *elF4E*, and *c-MYC*) in U937 and MV4-11 cells upon the treatment (DMSO, KPT-330, AZA, and combo). (**E**,**F**) Western blot data to show the changes of the key genes (XPO1, elF4E, and c-MYC) in U937 and MV4-11 upon the treatment (DMSO, KPT-330, AZA, and combo). * *p* < 0.05, ** *p* < 0.01, *** *p* < 0.001.

To investigate the underlying mechanisms of synergy, we performed RNA-seq analysis on U937 cells administrated with 0.7 μM AZA to identify 4655 differentially expressed genes (DEGs). *MYC* is among the top 5 DEGs with AZA administration, and gene set enrichment analysis (GSEA) revealed that *MYC* and the apoptotic pathway were enriched in DEGs (Figure 3A,B). Expression of XPO1, elF4E, and c-MYC is significantly down-regulated in U937 and MV4-11 cells upon the combo treatment compared to either single drug control in mRNA level (Figure 3C,D) and protein level (Figure 3E,F). Kyoto Encyclopedia of Genes and Genomes (KEGG) analysis revealed that DEGs are abundant in the MAPK signaling network, FoxO signaling route, and TNF signaling circuit (Figure S5). GO enrichment analysis showed that the DEGs were primarily enriched in apoptosis, cell cycle progression, and membrane functions (Figure S5).

### 2.4. c-MYC-Dependence on the Combination-Mediated Proliferation Arrest and Apoptosis in U937 Cells

**Figure 4:**
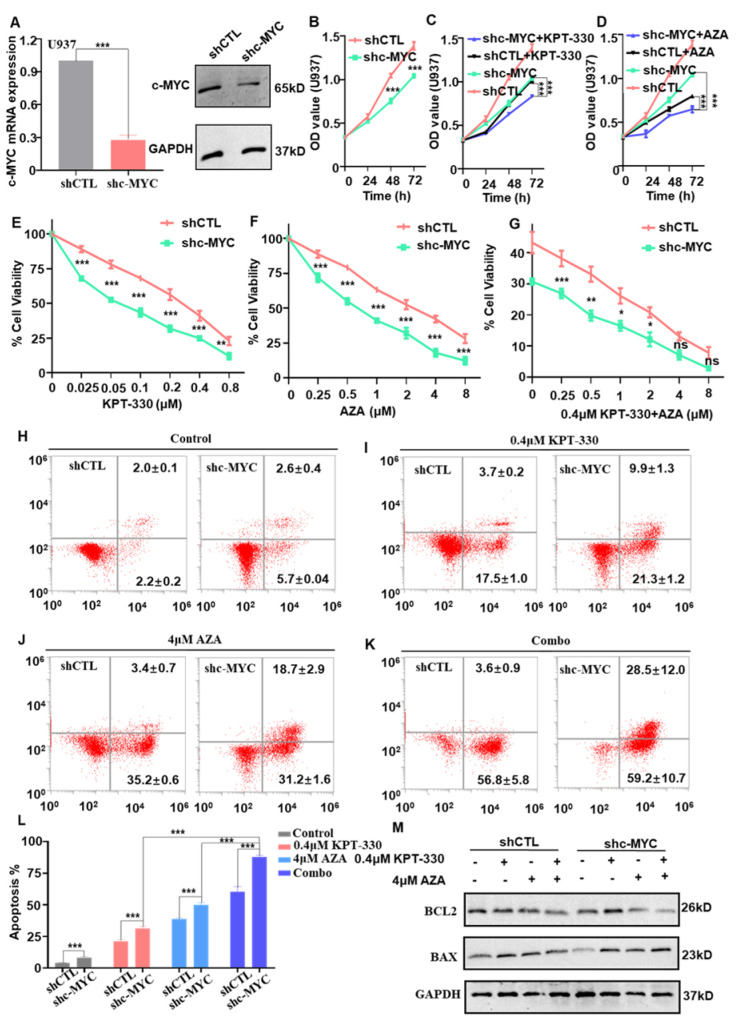
Effect of c-MYC knockdown on the combination-mediated proliferation in U937 cells. (**A**) c-MYC was efficiently knocked down in the U937 cell by qPCR and western blot. (**B**) c-MYC knockdown (shc-MYC) inhibits cell proliferation arrest compared to that of scramble shRNA (shCTL) in U937 cells. (**C**,**D**) The effect of c-MYC knockdown by shRNA with XPO1 inhibitor or AZA on cell proliferation progression was analyzed by CCK-8 assay. Cells were treated with 0.1 μM KPT-330 or 1 μM AZA for 0–72 h for cell proliferation assay. (**E**–**G**) c-MYC knockdown facilitated the effect of KPT-330, AZA, and combo on cell proliferation arrest in U937 cells compared to shCTL. (**H**–**K**) The effect of shc-MYC facilitated the effect of KPT-330, AZA, and combo on cell apoptosis in U937 cells compared to shCTL. (**L**) The statistical chart shows the apoptosis effect of c-MYC knockdown by KPT-330, AZA, and combo in shCTL and shc-MYC cells. (**M**) Western blot data to show the changes of the apoptosis-related genes (BCL2 and BAX) in combo with shCTL and shc-MYC cells. * *p* < 0.05, ** *p* < 0.01, *** *p* < 0.001, ns: *p* > 0.05.

*c-MYC* is a well-documented oncogene in hematological malignancies [33,34]. To investigate the biological function of *c-MYC* in AML cells, we knocked c-MYC down in U937 cells with c-MYC shRNA. RT-qPCR and western blot data showed that c-MYC was efficiently knocked down in U937 cells (Figure 4A). In U937 cells, c-MYC knockdown (shc-MYC) dramatically inhibits cell growth compared to scrambling shRNA (shCTL) (Figure 4B). C-MYC knockdown significantly sensitized the effect of XPO1 inhibitor (Figure 4C) or AZA (Figure 4D) on cell proliferation arrest in a time-dependent manner. Moreover, c-MYC knockdown intensified the impact of KPT-330 (Figure 4E), AZA (Figure 4F), and the combination (Figure 4G) on cell proliferation arrest relative to shCTL in a dose-dependent manner. These findings demonstrated the critical role of c-MYC in the synergistic combination impact on cell growth arrest.

Moreover, c-MYC knockdown significantly enhanced U937 apoptosis compared to shCTL (Figure 4H), and it significantly sensitized the apoptotic effect of KPT-330 (Figure 4I), AZA (Figure 4J), and combo (Figure 4K) compared to that of the shCTL control. The quantitative data showed that the combo had a significantly higher effect in c-MYC knockdown cells than single drug control (Figure 4L). The BCL-2 expression decreased, and BAX expression increased dramatically upon the drug treatment (DMSO, KPT-330, AZA, combo) in the c-MYC knockdown cells and shCTL cells (Figure 4M). These data revealed that c-MYC knockdown promotes combination-mediated apoptosis in AML cells. 

### 2.5. XPO1/eIF4E Was Up-Regulated in AML Patients and Expression Was Associated with a Worse Prognosis

**Figure 5:**
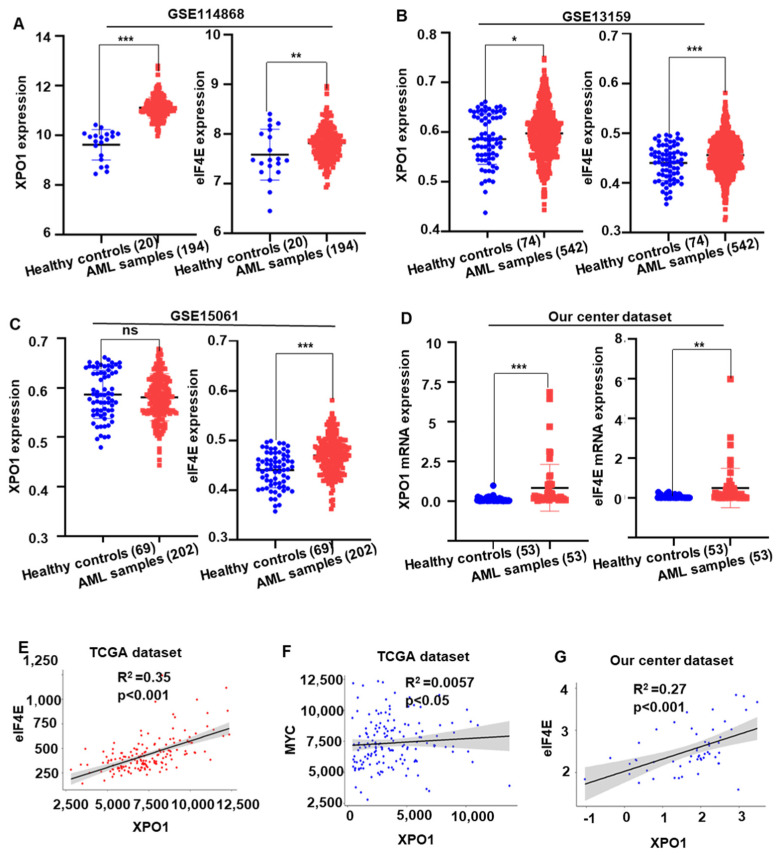
*XPO1/eIF4E* was highly expressed and correlated in AML patients. (**A**) Gene expression of *XPO1* and *eIF4E* in AML patients (n = 194) and healthy donors (n = 20) extracted from the GEO (GSE11486) database. (**B**) Gene expression of *XPO1* and *eIF4E* in AML patients (n = 542) and healthy donors (n = 74) from the GEO (GSE13159) database. (**C**) Expression of the *XPO1* and *eIF4E* genes in AML patients (n = 202) and healthy donors (n = 69) from the GEO (GSE15061) database. (**D**) In our institute’s cohort research, blood samples from AML patients (n = 53) express greater levels of *XPO1* and *eIF4E* than samples from healthy donors (n = 53). (**E**−**G**) the association between *XPO1* and *eIF4E* in the TCGA dataset and the dataset from our institute. * *p* < 0.05, ** *p* < 0.01, *** *p* < 0.001, ns: *p* > 0.05.

To understand the oncogenic role of *XPO1* and *eIF4E*, the mRNA level of *XPO1* and *eIF4E* was tested in AML patients from the GEO (GSE11486; GSE13159 and GSE15061) database. Results showed that the *XPO1* and *eIF4E* are significantly highly expressed in AML patients compared to the normal health donors (Figure 5A–C). We evaluated the mRNA levels of *XPO1* and *eIF4E* in 53 AML patients and 53 healthy donors. Results showed that the mRNA level of *XPO1* and *eIF4E* was significantly elevated in AML samples compared to that of healthy donor controls (Figure 5D). In the *XPO1* target genes, we found that *eIF4E* and *c-MYC* had a highly positive correlation with *XPO1* in TCGA dataset, respectively (all *p* < 0.05, Figure 5E,F). In addition, the same trend also existed in our center AML data, *XPO1* was positively correlated with *eIF4E* (*p* < 0.001, Figure 5G). It implied that *XPO1* and *eIF4E* are important in AML patients, and more clinical characteristics need to be studied. Then, in our center dataset, we divided the AML patients into the *XPO1* high (top 25%, n = 13) and *XPO1* low (bottom 75%, n = 40) expression groups, as well as the *eIF4E* high (top 25%, n = 13) and *eIF4E* low (bottom 75%, n = 40) expression groups, based on the *XPO1* and *eIF4E* mRNA intensity, respectively. The correlation of characteristics of AML patients with *XPO1* and *eIF4E* expression are summarized in Table S1. There was no difference in clinical characteristics between the high and low-expression groups of *XPO1*. The *eIF4E* high group tended to have more *FLT3* (2/11 vs. 3/37) and *TP53* (2/38 vs. 2/11) mutations than the *eIF4E* low expression group.

**Figure 6:**
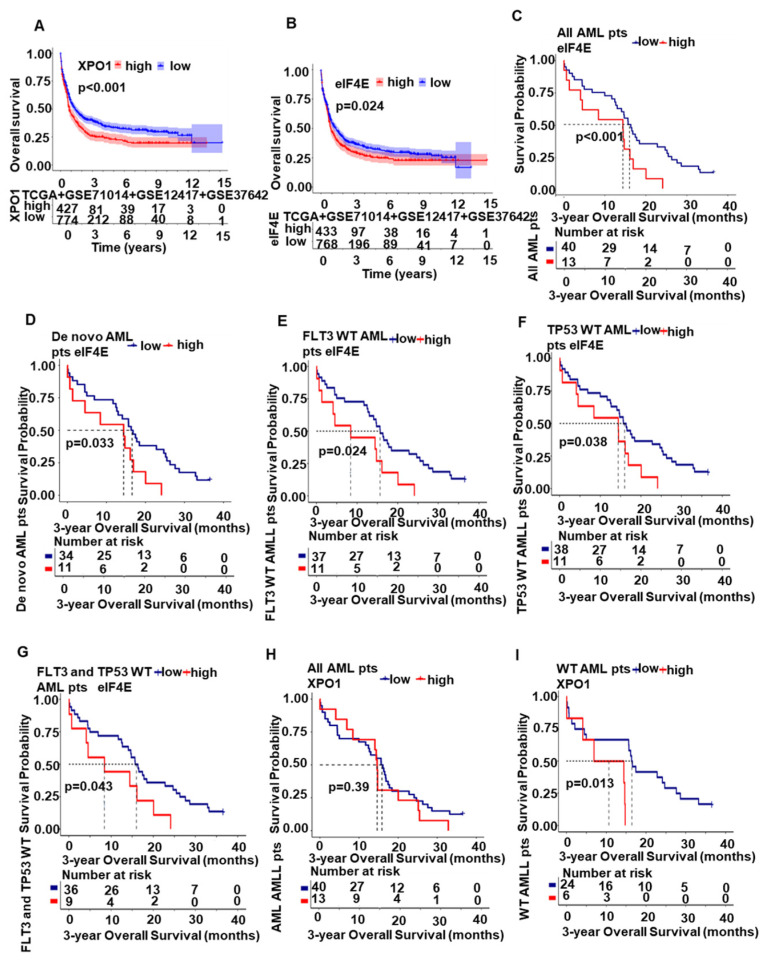
*XPO1*/*eIF4E* was associated with a worse prognosis. (**A**) Overall survival of AML patients with high *XPO1* expression (n = 427) and low *XPO1* expression (n = 774) based on the combined database (TCGA + GSE71014+ GSE12417 + GSE37642). (**B**) Overall survival of AML patients with high *eIF4E* expression (n = 433) and those with low *eIF4E* expression (n = 768) according to the combined database (TCGA + GSE71014 + GSE12417 + GSE37642). (**C**,**D**) The 3-year overall survival from our center AML cohort and the de novo cohort of high *eIF4E* expression and that of low *XPO1* expression. (**E**–**G**) *FLT3* wild-type patients, *TP53* wild-type patients, and *FLT3* and *TP53* wild-type patients showed worse 3-year OS in the high *eIF4E* group compared to the low *eIF4E* group. (**H**) A similar but nonsignificant trend was found in *XPO1* expression groups in all AML patients of the 3-year OS. (**I**) The 3-year OS of the *XPO1* high group was significantly shorter than that of the low-expression group without the following mutations: *FLT3*, *TP53*, *IDH1/2*, *DNMT3A*, and *TET2*.

Furthermore, we found that the patients with high *XPO1* expression (Figure 6A) and high *eIF4E* expression (Figure 6B) have significantly short overall survival (OS) compared with that with low *XPO1* and *eIF4E* expression. Besides, in our center, patients with high *eIF4E* significantly had shorter 3-year OS compared to that with low *eIF4E* in all AML patient cohorts (Figure 6C, *p* < 0.05) and the de novo cohort (Figure 6D, *p* < 0.05). The *eIF4E* high group had poor 3-year OS in *FLT3* wild-type patients (Figure 6E, *p* < 0.05), *TP53* wild-type patients (Figure 6F, *p* < 0.05), and *FLT3* and *TP53* wild-type patients (Figure 6G, *p* < 0.05) compared to the low group. A similar but nonsignificant trend was seen in the *XPO1* expression groups among all AML patients with a 3-year OS (Figure 6H, *p* = 0.39). However, when screening patients without the following mutations: *FLT3*, *TP53*, *IDH1/2*, *DNMT3A*, and *TET2*, the 3-year OS of the *XPO1* high group was significantly shorter than that of the low-expression group (Figure 6I, *p* < 0.05). These data reveal that *XPO1*/*eIF4E* high expression has oncogenic roles in AML.

Furthermore, we found that the patients with high *XPO1* expression (Figure 6A) and high *eIF4E* expression (Figure 6B) have significantly short overall survival (OS) compared with that with low *XPO1* and *eIF4E* expression. In our institution, patients with high *eIF4E* had significantly shorter 3-year OS than those with low *eIF4E* in all AML patient cohorts (Figure 6C, *p* < 0.05) and the de novo cohort (Figure 6D, *p* < 0.05). The *eIF4E* high group had poor 3-year OS in *FLT3* wild-type patients (Figure 6E, *p* < 0.05), *TP53* wild-type patients (Figure 6F, *p* < 0.05), and *FLT3* and *TP53* wild-type patients (Figure 6G, *p* < 0.05) compared to the low group. A similar but nonsignificant trend was seen in the *XPO1* expression groups among all AML patients with a 3-year OS (Figure 6H, *p* = 0.39). However, when screening patients without the following mutations: *FLT3*, *TP53*, *IDH1/2*, *DNMT3A*, and *TET2*, the 3-year OS of the *XPO1* high group was significantly shorter than that of the low-expression group (Figure 6I, *p* < 0.05). These data reveal that *XPO1*/*eIF4E* high expression has oncogenic roles in AML.

### 2.6. Synergistic Effect of KPT-330 with AZA on Cell Growth Arrest in Primary Cells from the AML Patients

**Figure 7:**
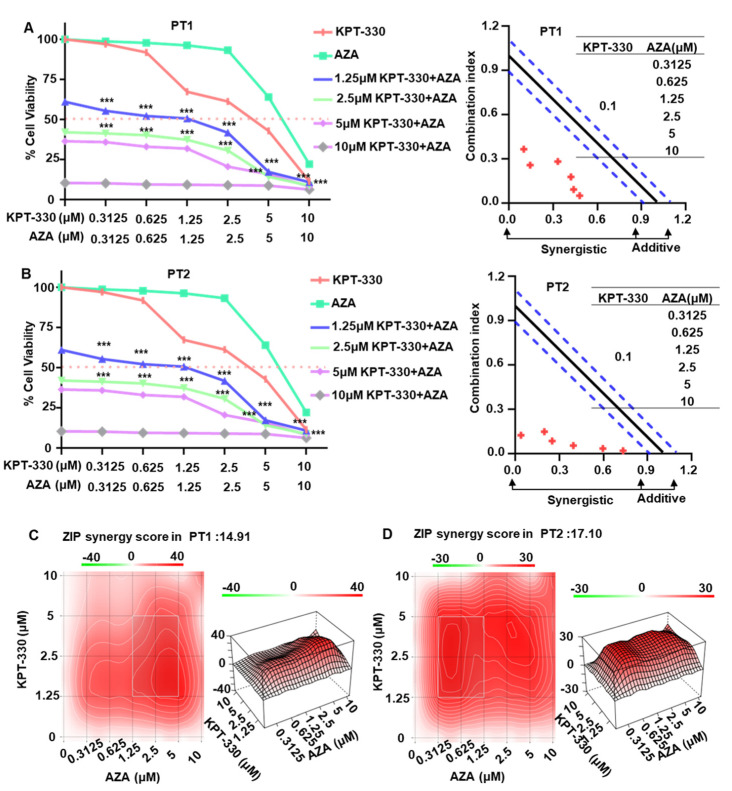
Synergistic effect of KPT-330 with AZA on cell growth arrest in primary cells from the AML patients. Effect of drug treatment (DMSO, KPT-330, AZA, and combo) on the cell growth arrest and CalcuSyn analysis in primary cells from AML patient 1 (PT1, (**A**)). Effect of drug treatment (DMSO, KPT-330, AZA, and combo) on the cell growth arrest and CalcuSyn analysis in primary cells from AML patient 2 (PT2, (**B**)). Bliss analysis for the synergistic effect in PT1 (**C**) and PT2 (**D**) using the SynergyFinder Web application. Synergy score: <−10, antagonistic; from −10 to 10, additive; >10, synergistic. *** *p* < 0.001.

Next, we explored the effect of KPT-330 combined with AZA on cell proliferation arrest of primary cells from two AML patients (PT1 and PT2). Results showed that the fixed doses (1.25 μM, 2.5 μM, 5 μM, 10 μM) of KPT-330 significantly enhanced the dose-dependent effect of AZA on cell proliferation arrest in the primary cells from PT1 (Figure 7A) and PT2 (Figure 7B). Both CalcuSyn and ZIP analysis showed significant synergistic effects and synergistic doses of KPT-330 and AZA in the cells (Figure 7A–D). These results suggested that the combination of KPT-330 and AZA has a synergistic impact on the inhibition of cell growth in primary AML cells.

### 2.7. Synergistic Effect of KPT-330 with AZA on Cell Growth Arrest in Primary Cells from the AML Patients

**Figure 8:**
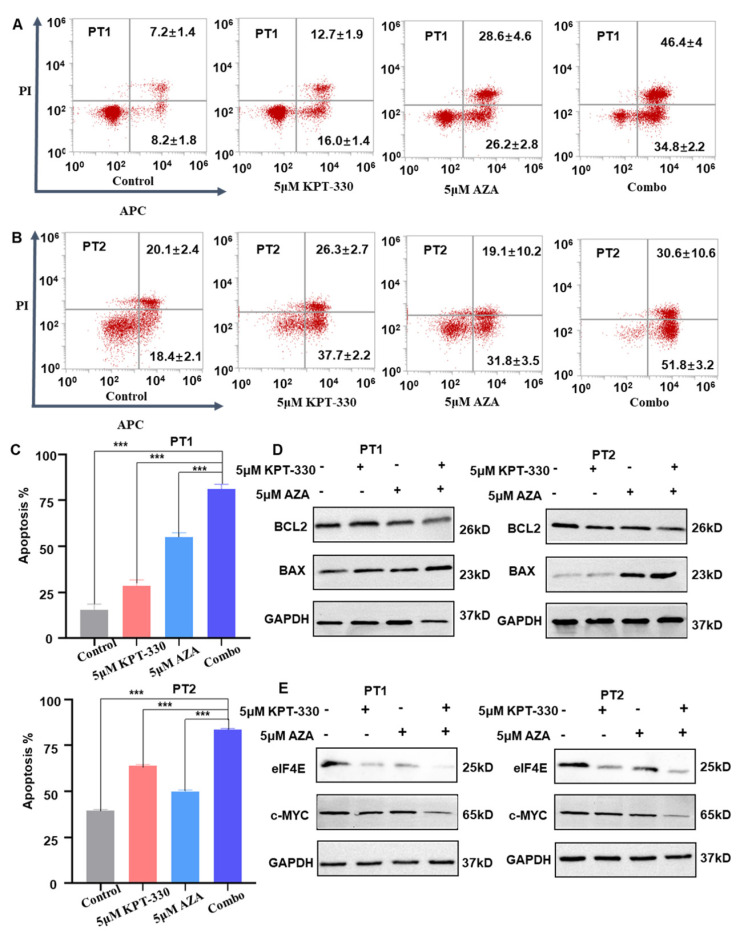
Synergistic effect of KPT-330 with AZA on apoptosis in primary cells from the AML patients. (**A**) Effect of drug treatment (DMSO, KPT-330, AZA, and combo) on apoptosis in primary cells from AML patient 1 (PT1). (**B**) Effect of drug treatment (DMSO, KPT-330, AZA, and combo) on apoptosis in primary cells from AML patient 2 (PT2). (**C**) Quantitative bar chart for PT1 and PT2. (**D**) Effect of drug treatment (DMSO, KPT-330, AZA, and combo) on apoptosis-related protein (BCL2 and BAX) in primary cells from AML PT1 and PT2. (**E**) Effect of drug treatment (DMSO, KPT-330, AZA, and combo) on XPO1, elF4, and c-MYC in primary cells from AML patient PT1 and PT2. *** *p* < 0.001.

Moreover, combining KPT-330 with AZA significantly increased the apoptosis in the primary cells compared to either each single drug control (Figure 8A–C). Consistently, the pro-apoptotic BAX protein was enhanced considerably, but the anti-apoptotic BCL-2 protein was significantly reduced upon the combination treatment compared to either single drug control in the cells (Figure 8D). The mRNA level of the apoptosis-related genes (*BCL2*, *BAX*, and *DDIT3*) showed a similar change upon the drug treatment in U937, MV4-11, and THP-1 cells (Figure S6A,B). These results indicated that combining XPO1 inhibition with AZA synergizes apoptosis in primary cells from AML patients. 

Moreover, we detected the influence of KPT-330 and AZA on the expression of c-MYC and eIF4E in the primary cells. Results showed that the combination down-regulated c-MYC and eIF4E expression at the protein level (Figure 8E). These results indicated that combining XPO1 inhibitor with AZA exerts its synergistic effect by inhibiting the XPO1/eIF4E/c-MYC signaling in AML. In addition, the molecular mechanism model underlying the synergistic effect of the combination of XPO1 inhibitor with AZA was depicted in Figure S7. 

## 3. Discussion

AML is a relatively common hematological malignancy characterized by a low survival rate and inadequate available therapies [35,36]. Aberrant DNA methylation is a common feature in AML, and although AZA, a targeted demethylation inhibitor AZA, has been used in AML, some patients do not respond to AZA, and most treatment-responsive patients eventually acquire resistance [16,37]. Targeted therapy has emerged as a promising treatment strategy for chemotherapy-resistant AML, where combinations of venetoclax and DNA methyltransferase inhibitors have proven successful examples [38,39]. However, myelosuppression can be a significant side effect of this treatment [40], emphasizing the need for alternative regimens that are safer and more effective. We sought to test the synergistic lethality of the XPO1 inhibitor KPT-330 and AZA in AML cell lines and primary AML cells from patients by reducing c-MYC expression to mediate apoptosis. In this study, we provide encouraging evidence that AZA acts in conjunction with KPT-330 in the treatment of preclinical AML.

Our study found that the combination of AZA and KPT-330 resulted in enhanced apoptosis compared with drug therapy alone. Apoptosis is crucial in preventing cancer development and is mediated by proteins such as BCL-2 and BAX, which leads to rapid cell death with unique biochemical and morphological characteristics [41]. In the present study, Our results showed that combined treatment with KPT-330 and AZA increased BAX expression and decreased anti-apoptotic BCL-2 expression, promoting apoptosis, which contributes to apoptosis. Furthermore, CCK-8 results confirmed the synergistic effects of the two compounds on AML cell proliferation, suggesting the potential use of KPT-330 in combination with AZA for treating AML patients. We further investigated the possible mechanisms by which KPT-330 and AZA caused cell proliferation arrest and apoptotic effect in AML cells.

One possible mechanism to explain the synergistic effect of KPT-330 in combination with AZA is their ability to alter c-MYC expression jointly. Our data suggested that *c-MYC* is at the top list of DEGs upon AZA treatment (Figure 3A), and gene set enrichment analysis (GSEA) indicated c-MYC targeted signaling and apoptosis pathway are enriched (Figure 3B). Then, the mRNA level and protein level changes of the key genes c-MYC in U937 and MV4-11 cells were down-regulated upon the treatment (DMSO, KPT330, AZA, and combo; Figure 3C–F). In addition, the combination therapy can synergistically modulate the significant reduction of c-MYC protein expression in 2 AML patients, compared with monotherapy (Figure 8E).

Various ontogenetic abnormalities in AML are closely associated with the dysregulation of c-MYC expression, such as c-MYC amplification [42,43]. Notably, the inhibiter of c-MYC target gene transcription by modulating c-MYC target genes resulted in the gene expression silencing of c-MYC and caused cell proliferation arrest [44], which is well confirmed by our results (Figure 4A,B). As the c-MYC expression is dysregulated in genomic subtypes of AML [45,46], it may be an attractive target for targeted antileukemic therapy. c-MYC is a key transcription factor that alters the expression of various oncogenes, and c-MYC also plays a crucial role in AML cell survival [47,48]. Then, our data indicated that downregulation of c-MYC in U937 cells increased sensitivity to single drug KPT-330 or AZA and was also sensitized to combination-mediated proliferation compared to shCTL (Figure 4E–G). RT-qPCR and western blot data showed that c-MYC knockdown not only upregulated the expression of apoptotic proteins BAX but also decreased the expression of BCL-2 level in U937 cells, resulting in the activation of the classical apoptotic pathway (Figure 4M).

KPT-330, a novel group of small molecule inhibitors, focuses on XPO1, a crucial nucleocytoplasmic transporter accountable for the nuclear export of significant tumor suppressor proteins and growth regulators like p53, PTEN, and FOXO [46]. Moreover, XPO1 is responsible for exporting the translation initiation factor eIF4E, which influences the translation of messenger RNAs for vital oncogenes such as c-MYC, BCL2, and BCL6 [49,50]. Inhibition of XPO1 prevents eIF4E from enhancing c-MYC translation; thus, a series of alternative therapeutic pathways work by disrupting c-MYC signaling and subsequently antagonizing its oncogenic activity [51,52]. As a result, the combination of these drugs could potentially reduce the mRNA and protein levels of XPO1, eIF4E, and c-MYC. Given the critical function of XPO1 in controlling these crucial proteins, it is not unexpected that an elevation in XPO1 and eIF4E expression is frequently linked to an unfavorable prognosis in both solid and hematologic cancers [53,54,55]. Our data indicated that XPO1/eIF4E was overexpressed in AML patients and accompanied by a worse prognosis. 

The presence of XPO1 played a crucial role in regulating the stability of oncogenetic mRNA, including eIF4E and c-MYC, under such conditions [56,57]. It is believed that the degradation process is dependent on XPO1 and occurs in response to treatment with AZA and Selinexor. As to the mechanism of the XPO1 degradation, it will be illustrated in further study.

Conclusively, our study demonstrated that the combination of KPT-330 and AZA synergistically induced proliferation and promoted apoptosis in AML cells. In addition, inhibition of XPO1 resulted in a significant downregulate of the expression of eIF4E/c-MYC, which plays a key role in apoptosis induced by combination therapy. Subsequent research should demonstrate the effectiveness of such treatment plans in vivo before their clinical application. Therefore, KPT-330 combined with AZA may be a potential treatment for AML patients. Our results provide preclinical evidence and clear direction for clinical trials combining KPT-330 and AZA in AML patients. Based on the present data, a clinical trial of KPT-330 plus AZA in AML is under registration and will be performed.

## 4. Materials and Methods

### 4.1. Samples from AML Patients 

We collected bone marrow samples from 53 newly diagnosed AML patients and 53 healthy controls between 4 March 2016, and 30 January 2022, at Zhongda Hospital (affiliated with the Southeast University). Written informed consent from all enrolled patients was collected according to the tenets of the Helsinki Declaration, and the study was approved by the Ethics Committee (Zhongda Hospital, Southeast University) (2019ZD-SYLL121-P01, 20 August 2019; 2017ZDSYLL067-P01, 10 August 2017; 2016ZDKYSB062, 4 March 2016) [35]. Mononuclear cells were isolated from the bone marrow samples using Ficoll.

We obtained primary cells from two recently diagnosed AML patients (referred to as PT1 and PT2) who had high leukocyte counts via leukapheresis. To enrich mononuclear cells, we used Ficoll and then lysed them with a red blood cell lysis buffer from Biosharp, China. We subsequently cultured the primary AML cells in RPMI 1640 containing 10% FBS to use in different experiments.

### 4.2. Cell Lines 

We obtained AML cell lines U937 (CRL-1593.2), MV4-11 (CRL-9691), and THP-1 (TIB-202) from the American Type Culture Collection (ATCC, Philadelphia, PA, USA). The cells were cultured and passaged every two to three days to maintain exponential growth. We used thawed cells between 10 and 50 passages for subsequent cell experiments. IMDM (Iscove’s Modified Dulbecco Medium, Shanghai, China) was used to culture MV4-11 cells, whereas U937 and THP-1 cells were cultured in RPMI 1640 supplemented with 10% fetal bovine serum, 100 U/mL penicillin, and 100 μg/mL streptomycin. All the cell lines were kept at 37 °C and in a 5% CO_2_ atmosphere.

### 4.3. Reagents 

Selleck Chemicals (Shanghai, China) provided us with KPT-330 (Cat. No S7252) and AZA (Cat. No S1782) for our in vitro cell experiments. To dissolve both compounds, we used anhydrous dimethyl sulfoxide (DMSO). For flow cytometry experiments, we procured mouse anti-human monoclonal antibodies and apoptosis antibodies (BD bioscience, San Jose, CA, USA), including anti-CD34, anti-CD45, APC, PI, and FITC antibodies. The shRNA plasmid for c-MYC was obtained from Corues Biotechnology (Nanjing, China), and T101 (Vazyme, Nanjing, China) was used for transfection.

### 4.4. Cell Proliferation Assay

We used the CCK-8 assay, following the manufacturer’s instructions, to evaluate cell proliferation after treatment with AZA or KPT-330. Initially, cells were seeded in 96-well plates with 4 × 10^5^ cells per well in 50 µL of the growth medium, followed by the addition of 50 µL of drug medium at seven serially diluted concentrations to each well. After 24 or 48 h of incubation, 10 µL of CCK-8 test solution per well was added, and the plates were incubated for an additional 2 to 4 h before measuring absorbance using an ELx800 plate reader (BioTek, Shoreline, WA, USA) at 544 nm excitation and 590 nm emission wavelengths. We normalized relative cell viability to DMSO-treated wells and determined the IC50 value of AZA or KPT-330 as the concentration required to inhibit cell growth by 50%. To evaluate synergies between AZA and KPT-330, the combination index (CI) was calculated using the Compusyn software (ComboSyn Inc., Paramus, NJ, USA). We defined an additive effect as a CI value of 1, synergism as a CI value less than 1, and antagonism as a CI value greater than 1. We also performed a zero interaction potency (ZIP) score analysis to assess synergistic and antagonistic doses using a combination of Loewe and Bliss modeling theories. This analysis considered the entire dose-response matrix data set.

### 4.5. Apoptosis Assay by Flow Cytometry

The collected cells were washed twice with PBS and stained for apoptotic cells using the Apoptosis Detection Kit (556547; BD, CA, USA) as per the manufacturer’s instructions. For double staining of apoptotic cells, the AnnexinV/FITC kit (Thermo Scientific, Shanghai, China) was used with PI. The apoptotic cells were analyzed using the BDTM LSR II flow cytometry instrument (BD Biosciences, San Jose, CA, USA), and the data were analyzed using FlowJo v10 software (LLC, Ashland, OR, USA).

### 4.6. Cell Cycle Test

The cells were harvested and washed twice with cold phosphate-buffered saline (PBS), then fixed with 70% ethanol at 4 °C for 24 h. Afterward, the cells were washed twice with PBS, stained with propidium iodide solution (BD, USA) for 15 min at room temperature, and analyzed on flow cytometry (ThermoFisher, Waltham, MA, USA). Each experiment was performed in triplicate, with each sample also being triplicated.

### 4.7. RNA-Seq Analysis

U937 AML cells were subjected to treatment with either AZA (0.7 µM) or vehicle for 48 h. We used the TRIzol reagent (Takara Bio, Shanghai, China) to extract total RNA from the cells. Subsequently, we sent the mRNA expression profiles of U937 AML cells that underwent treatment with AZA or vector to a testing company (Novegene, Nanjing, China) for high-throughput transcriptome sequencing. Using the DEseq2 R package, we identified the differentially expressed genes (DEGs) between the two groups. Genes with their log2FoldChange over 1.5 and a *p*-value less than 0.05 were considered as differentially expressed, and the absolute value of the log2FoldChange was used for ranking. Using the cluster profiler R package, we determined the statistical enrichment of differentially expressed genes (DEGs) in GO and KEGG pathways.

### 4.8. Real-Time Quantitative PCR (RT-qPCR) 

Samples were subjected to TRIzol RNA extraction, followed by the preparation of complementary DNA for target gene detection using RT-qPCR, as described previously [35,58]. The following primer pairs were used for RT-qPCR analysis: *GAPDH*: 5′ GTCTCCTCTGACTTCAACAGCG 3′ and 5′ ACCACCCTGTTGCTGTAGCCAA 3′; *DDIT3*: 5′ GAGAATGAAAGG-AAAGTGGCAC 3′ and 5′ ATTCACCATTCGGTCAATCAGA 3′; *BCL2*: 5′ GAAGGTTTCCTCGTCCCTGG 3′ and 5′ CTGTGTTGAAACAGGCCACG 3′; *BAX*: 5′ TGATGGACGGTCCGGG 3′ and 5′ GGAAAAAGACCTCTCGGG GG 3′; *XPO1*: 5′ GTCA-GCTGCTTGATTTCAGCC 3′ and 5′ TCCTTCGCACTGGTTCCTTG 3′; *eIF4E*: 5′ CA-TGTTGGACCGACCTCCC 3′ and 5′ ACCAGAGTGCCCATCTGTTC 3′; *c-MYC*: 5′ CATCAGCACACTACGCAGC 3′ and 5′ GCTGGTGCATTTTCGGTTGT 3′. The PCR procedure involved an initial incubation at 95 °C for 5 min, followed by 40 cycles of incubation at 95 °C for 45 s, 56 °C for 30 s, and 72 °C for 45 s. The process concluded with a final extension at 72 °C for 10 min [58]. We used the 2^−ΔΔCt^ method to calculate the relative expression levels of the genes.

### 4.9. Western Blot

Protein levels in U937, MV4-11, or primary cell extracts were assessed by Western blot assay to determine the effects of 2 μM AZA, 0.4 μM KPT-330, and their combination. The protein samples were transferred onto nitrocellulose membranes and then incubated with primary antibodies diluted at 1:1000 concentration. Subsequently, we used secondary antibodies (horseradish peroxidase linked anti-rabbit IgG) for further incubation. The standard Western blot technique was followed, and detailed procedures have been described previously [35,58]. Primary antibodies used were GAPDH (#5174), eIF4E (#2067), c-MYC (#18,583), BCL-2 (#4223), and BAX (#5023) from Cell Signaling Technology (Shanghai, China). The GAPDH antibody was used as an internal control.

### 4.10. c-MYC shRNA Knockdown

Corues Biotechnology provided c-MYC-specific shRNA and control lentiviral particles. For lentiviral transduction, we inoculated 10,000 cells per well in 6-well tissue culture plates and infected them with lentiviral particles using the ExFect transfection reagent T101 for 48 h, following the instructions mentioned in the lipofectamine manual (Vazyme, Nanjing, China). After infection, U937 cells were maintained for 7–10 days and selected with 8 μg/mL puromycin. We determined the infection efficiency by analyzing the expression levels of c-MYC using RT-qPCR and western blot.

### 4.11. Bioinformatics Analysis of Public Databases

RNA sequencing data from healthy bone marrow (BM) and newly diagnosed/relapsed AML samples were obtained from GEO (GSE71014, GSE12417, and GSE37642) and TCGA database. The gene expression matrices were combined and batch effects were removed for data analysis using R (the Genome Analysis and Visualization Platform) (https://www.r-project.org/; accessed on 1 December 2022). The prognostic value of XPO1 and eIF4E for overall survival analysis in AML patients was conducted using R version 4.2.2.

### 4.12. Statistical Analysis

To analyze the quantitative data, we used IBM SPSS statistical software v26.0 for One-way ANOVA. Prism software v10.0 (GraphPad Software, San Diego, CA, USA) was used for data graphics. Student’s *t*-test of an independent sample was used for pair-wise comparisons to define differences. Mann–Whitney U test was used for continuous variables, and Fisher’s exact test was used for categorical variables. Simple linear regression and Spearman’s correlation analysis were used to clarify the correlations between XPO1 and eIF4E expression levels. The level of statistical significance was considered to be *p* < 0.05.

## Data Availability

The patient datasets for the current study are not publicly accessible by local health research ethics protocols; however, they may be available from the corresponding author.

## Supplementary Materials

The following supporting information can be downloaded at: https://www.mdpi.com/article/10.3390/ijms24076816/s1.
