# Supplementary material for: Azacitidine Is Synergistically Lethal with XPO1 Inhibitor Selinexor in Acute Myeloid Leukemia by Targeting XPO1/eIF4E/c-MYC Signaling
Source: Int J Mol Sci. 2023 Apr 6;24(7):6816. doi: 10.3390/ijms24076816 (PMC10094826; doi:10.3390/ijms24076816)
Supplement: Supplementary file 1 [file ijms-24-06816-s001.zip › ijms-2276446-supplementary.pdf]

Supplemental Figures and Tables

Azacitidine is synergistically lethal with XPO1 inhibitor Selinexor in acute myeloid leukemia by Targeting XPO1/eIF4E/c-MYC Signaling

Huideng Long[1#], Yue Hou[1#], Jun Li, Chunhua Song[2,3] and Zheng Ge[1*]

[1]Department of Hematology, Zhongda Hospital, School of Medicine, Southeast University, Institute of Hematology Southeast University, Nanjing 210009, China

[2]Hershey Medical Center, Pennsylvania State University Medical College, Hershey, PA17033, USA

[3]Division of Hematology, The Ohio State University Wexner Medical Center, the James Cancer Hospital, Columbus, OH 43210, USA

These authors contributed equally to the work.

Correspondence to:
Zheng Ge, M.D., Ph.D.
Department of Hematology, Zhongda Hospital, School of Medicine, Southeast University
Institute of Hematology Southeast University
87 Dingjiaqiao Street, Nanjing 210009, China,
Telephone: +86 25-83262468; Fax: +86 25-83262471
E-mail: zhengge@seu.edu.cn

ORCID: https://orcid.org/0000-0001-8028-1612

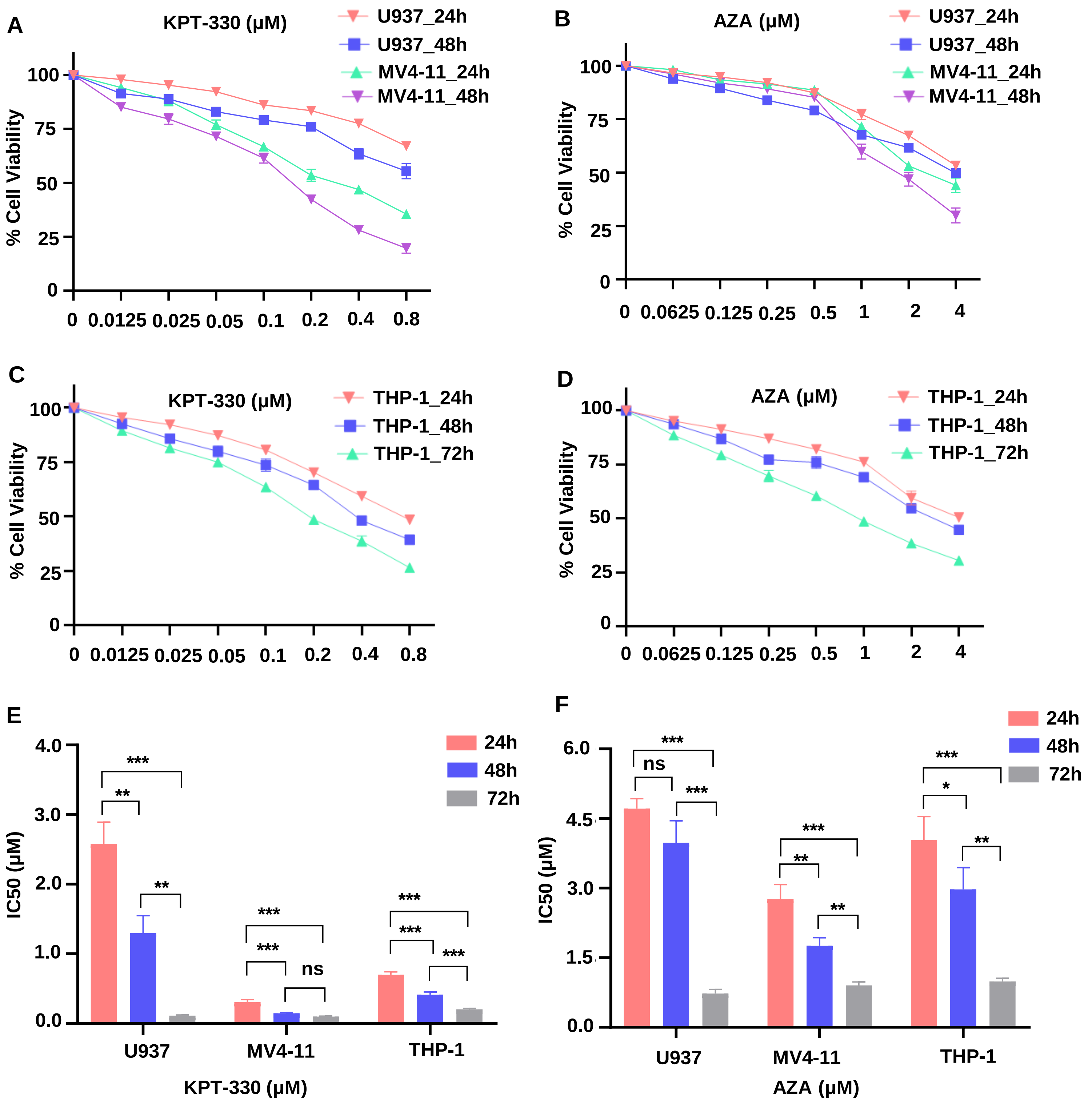

Figure S1 Growth inhibition of KPT-330 or AZA on AML cell lines. (A) Cell proliferation assay result (dose-dependent curve) for KPT-330 in U937 and MV4-11 for 24h and 48h. (B) Cell proliferation assay result (dose-dependent curve) for AZA in U937 and MV4-11 for 24h and 48h. (C) Cell proliferation assay result (dose-dependent curve) for KPT-330 in THP-1 for 24h, 48h and 72h. (D) Cell proliferation assay result (dose-dependent curve) for AZA in THP-1 for 24h, 48h and 72h. (E-F) IC50 values of three cell lines were calculated and an IC50 curve was observed based on the activity of KPT-330 or AZA. *$p < 0.05$, **$p < 0.01$, ***$p < 0.001$, ns:$p > 0.05$

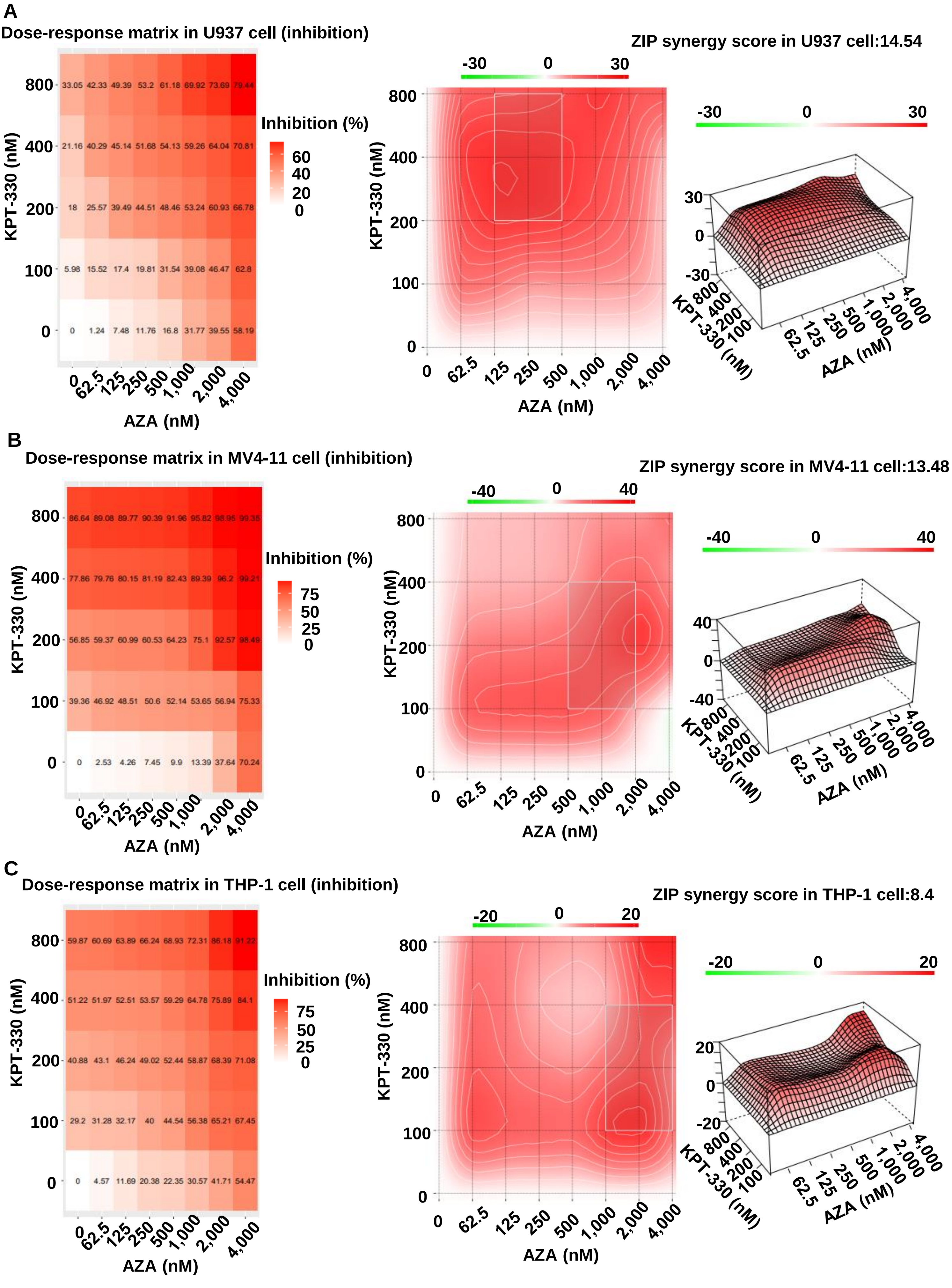

Figure S2 Bliss analysis of Synergy of the combination on cell proliferation arrest. (A-C) Cell viability was calculated for every dose combination of KPT-330 and AZA in U937 (A), MV4-11 (B) and THP-1 (C) cells using the SynergyFinder Web application. Synergy score: < -10, antagonistic; from -10 to10, additive; >10, synergistic.

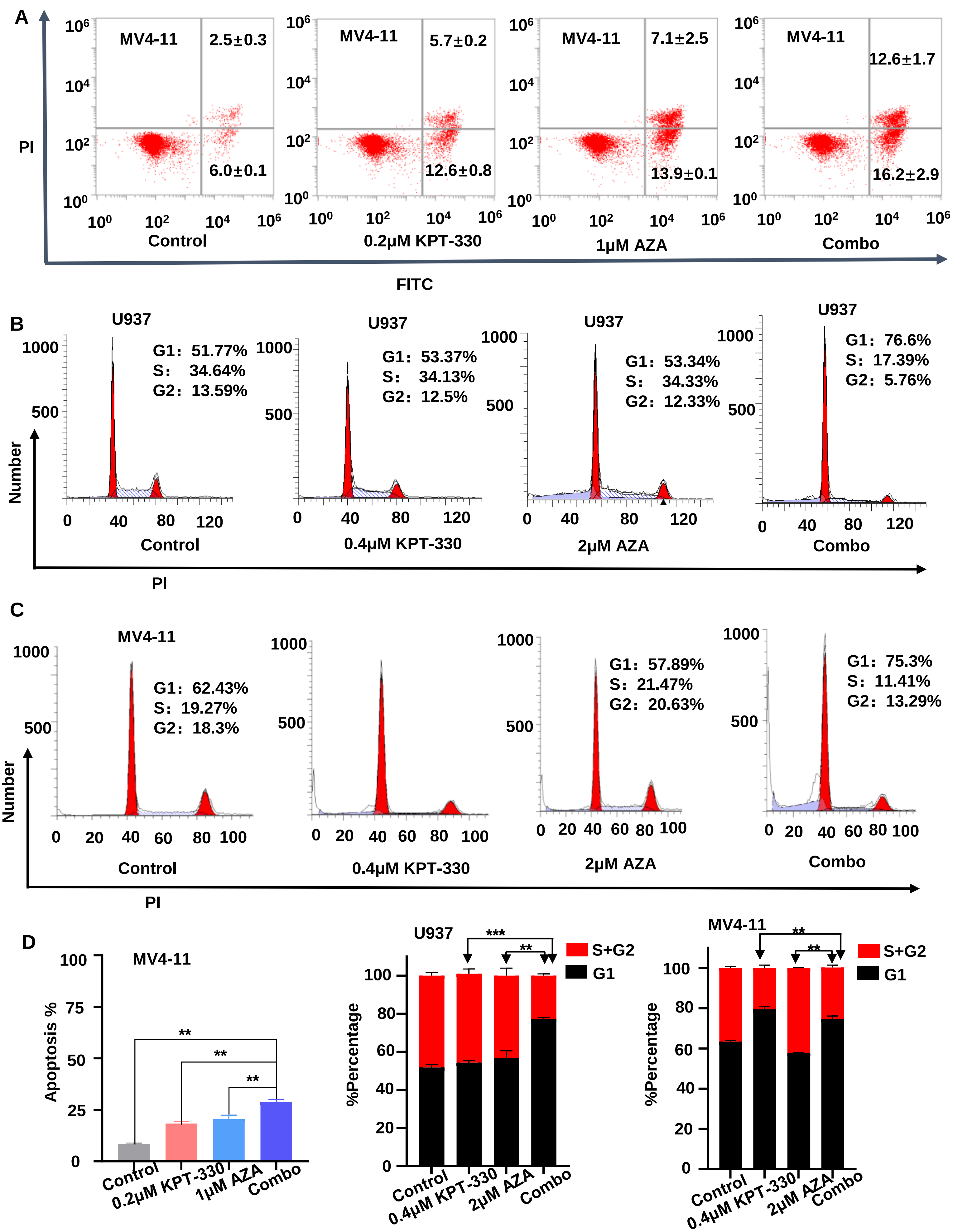

Figure S3 Effect of KPT-330 with AZA on apoptosis and cell cycle of AML cells by flow cytometry. (A) Effect of KPT-330 with AZA on apoptosis of MV4-11 cells. (B) Effect of KPT-330 with AZA on cell cycle of U937 cells. (C) Effect of KPT-330 with AZA on apoptosis of MV4-11 cells. (D) Quantitative bar figures in the cell lines (U937 and MV4-11). $^{**}p < 0.01$, $^{***}p < 0.001$

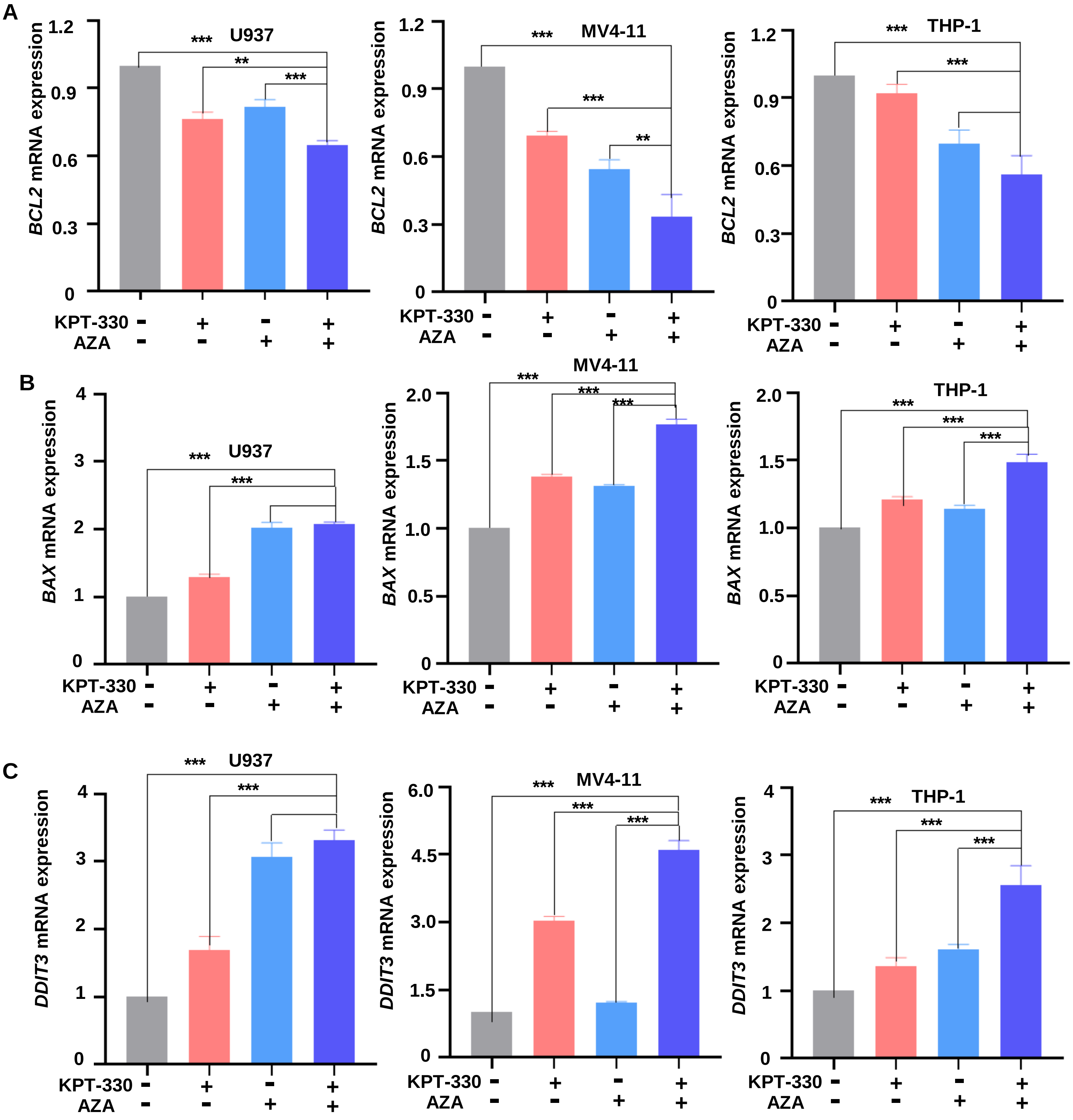

Figure S4 Change of mRNA level in the apoptosis-related genes (*BCL2, BAX and DDIT3*) upon the drug treatment in U937 (A), MV4-11 (B) and THP-1 (C) cells. **p < 0.01, ***p < 0.001

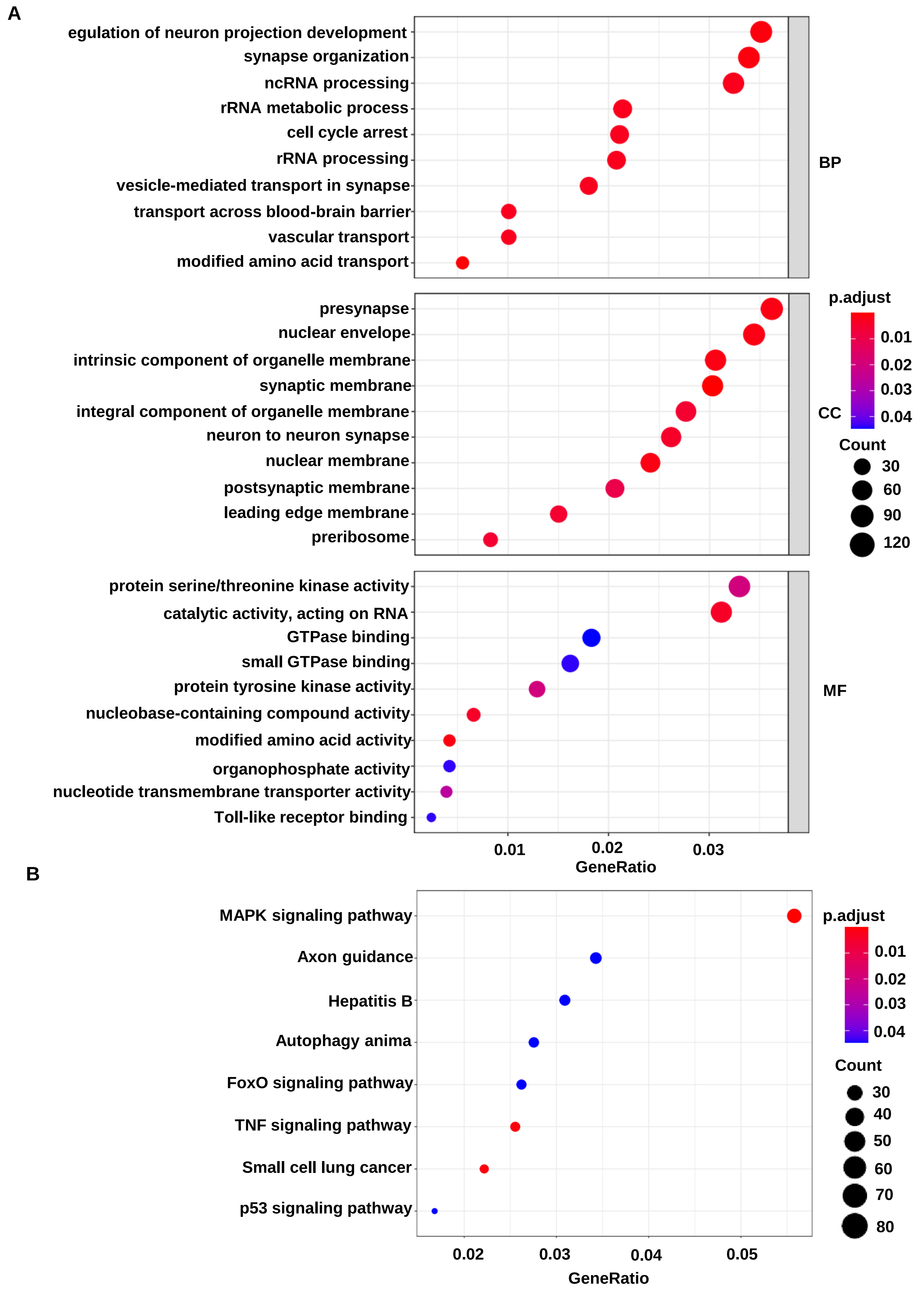

Figure S5 Transcriptome differential gene analysis to identify key signaling pathways by bioinformatics. (A) GO analysis figure. (B) KEGG analysis figure.

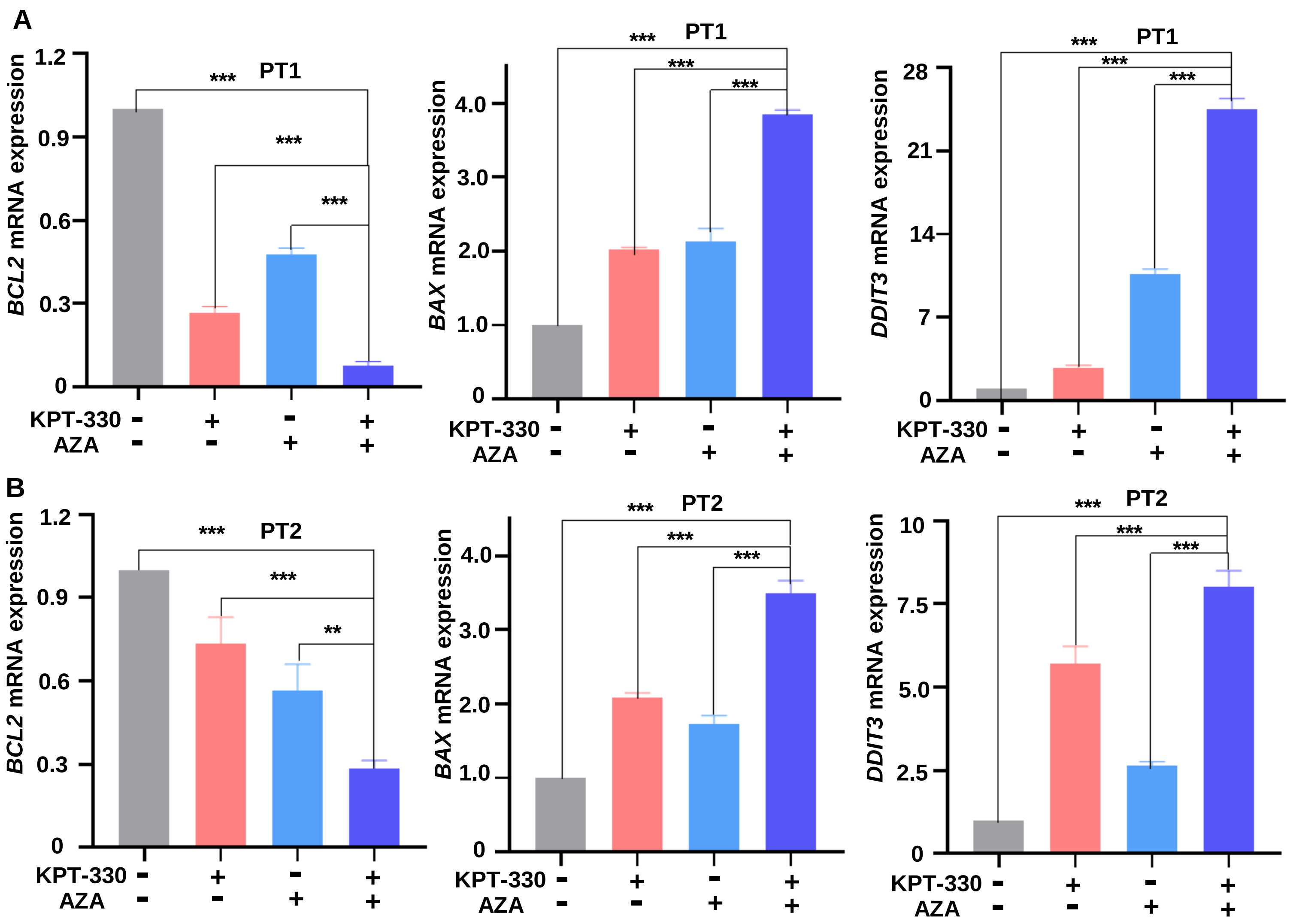

Figure S6 Change of mRNA level in the apoptosis-related genes (*BCL2, BAX and DDIT3*) were determined after drug exposure in PT1 (A) and PT2 (B). **$p < 0.01$, ***$p < 0.001$

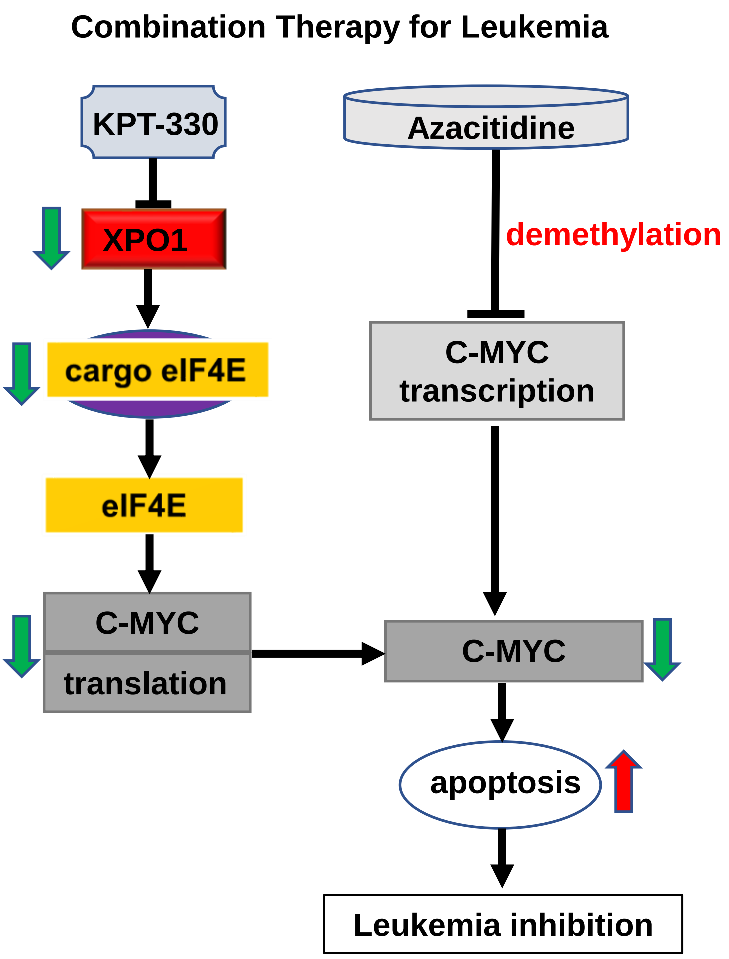

Figure S7 Anti-tumor mechanism model of the combination in AML.

Table S1. Correlation of characteristics of AML patients with *XPO1* and *eIF4E* expression in our center.

Characteristics	XPO1 expression (n=53)		P	eIF4E expression (n=53)		P
	low (n=40)	high (n=13)		low (n=40)	high (n=13)	
Sex, n (%)			0.534			0.338
Male	17(42.5)	7(53.8)		20(50)	4(30.8)	
Female	23(57.5)	6(46.2)		20(50)	9(69.2)	
FAB, n			0.597			0.256
M1	3	1		2	2	
M2	20	4		20	4	
M4	4	2		3	3	
M5	11	6		13	4	
NA	2	0		2	0	
Age, n			0.757			0.757
<60	19(47.5)	7(53.8)		19(47.5)	7(53.8)	
≥60	21(52.5)	6(46.2)		21(52.5)	6(46.2)	
BM blasts, %			0.385			0.620
Median(range)	60.6(23.5-96)	54.4(19-89)		64.25(19-96)	63.2(33.2-94.9)	
WBC(×109/L)			0.301			0.385
Median(range)	13.02(1.91-242.88)	4.8(0.4-73.97)		18.29(0.69-94.47)	33.34(0.4-242.88)	
Hb(g/L)			0.602			0.888
Median(range)	79(36-112)	70.5(36-152)		70.5(36-152)	71.5(42-112)	
ELN risk, n (%)			0.898			0.107
Favorable	8(20)	3(23.1)		10(25)	1(7.7)	
Intermediate/Poor	30(75)	9(69.2)		29(72.5)	10(76.9)	
NA	2(5)	1(7.7)		1(2.5)	2(15.4)	
Karyotype, n (%)			0.625			0.174
normal	22(55)	9(69.2)		22(55)	9(69.2)	
abnormal	15(37.5)	3(23.1)		16(40)	2(15.4)	
NA	3(7.5)	1(7.7)		2(5)	2(15.4)	
FLT3, n (%)			0.805			0.586
Wt	36(90)	12(92.3)		37(92.5)	11(84.6)	
Mut	4(10)	1(7.7)		3(7.5)	2(15.4)	
TP53, n (%)			0.982			0.249
Wt	37(92.5)	12(92.3)		38(95)	11(84.6)	
Mut	3(7.5)	1(7.7)		2(5)	2(15.4)	

There was no difference in clinical characteristics between the high and low-expression groups of *XPO1*. The *eIF4E* high group tended to have more *FLT3* (2/11 vs. 3/37) and *TP53* (2/11 vs. 2/38) mutations than the *eIF4E* low expression group.
